# Bilateral Lipid Keratopathy Treated with Staged Penetrating Keratoplasty: Restoration of Corneal Transparency and Visual Function

**DOI:** 10.3390/diagnostics16101551

**Published:** 2026-05-20

**Authors:** Wojciech Luboń, Mariola Dorecka

**Affiliations:** 1 Department of Ophthalmology, Faculty of Medical Sciences, Medical University of Silesia, 40-514 Katowice, Poland; mdorecka@sum.edu.pl; 2 Department of Ophthalmology, Professor K. Gibiński University Clinical Center, Medical University of Silesia, 40-514 Katowice, Poland

**Keywords:** lipid keratopathy, penetrating keratoplasty, corneal opacity, anterior segment optical coherence tomography, corneal transplantation, slit-lamp imaging, visual acuity, rare corneal disease

## Abstract

Lipid keratopathy is an uncommon corneal disorder characterized by stromal lipid deposition that may cause progressive corneal opacity and visual impairment. We report a case of advanced bilateral lipid keratopathy with severe visual-axis involvement. At presentation, best-corrected visual acuity (BCVA) was counting fingers in the right eye and 0.1 Snellen (1.0 logMAR) in the left eye. Slit-lamp examination and anterior segment optical coherence tomography (AS-OCT) demonstrated dense stromal lipid deposits involving the visual axis in both eyes. The patient underwent staged bilateral penetrating keratoplasty, with procedures performed three months apart. Postoperatively, corneal transparency improved in both eyes. At 6 months, BCVA was 0.5 Snellen (0.3 logMAR) in the right eye and 0.7 Snellen (0.15 logMAR) in the left eye. Residual visual limitation was attributed mainly to coexisting cataract, and sequential cataract surgery was planned. Together, the clinical photographs and AS-OCT scans illustrate an uncommon presentation of visually disabling bilateral lipid keratopathy, characterized by dense central stromal lipid deposition involving both visual axes and profound preoperative visual loss. The case is clinically noteworthy because it combines severe bilateral disease, close clinical–tomographic correlation, and sequential penetrating keratoplasty performed as a staged visual rehabilitation strategy, resulting in restoration of graft clarity and meaningful visual improvement during postoperative follow-up.

**Figure 1:**
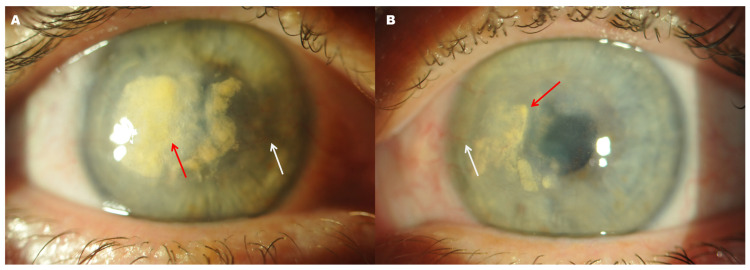
Preoperative slit-lamp photographs of bilateral lipid keratopathy in a 57-year-old woman. (**A**) Right eye showing dense yellow-white central stromal lipid deposition, marked corneal opacity, and near-complete obscuration of the pupil. (**B**) Left eye showing stromal lipid deposits with less confluent opacification and partial visibility of the pupillary margin. In both eyes, the deposits involved the visual axis, consistent with a severe reduction in BCVA at presentation: counting fingers in the right eye and 0.1 Snellen (1.0 logMAR) in the left eye. Red arrows indicate the largest and densest stromal lipid deposits, whereas white arrows indicate mild superficial peripheral corneal neovascularization, present bilaterally mainly in the nasal quadrants, without marked central vascular ingrowth. The patient had no history of ocular trauma, previous ocular surgery, recurrent keratitis, or systemic lipid disorder; therefore, in the absence of an identifiable precipitating ocular or systemic condition, the presentation was classified clinically as primary idiopathic lipid keratopathy. Preoperative B-scan ultrasonography did not reveal posterior segment pathology, supporting the cornea as the main cause of visual impairment. Lipid keratopathy is associated with stromal lipid accumulation, frequently in the context of corneal neovascularization or chronic inflammation; when visual-axis involvement is advanced, surgical intervention may be required to restore optical clarity [1,2].

**Figure 2:**
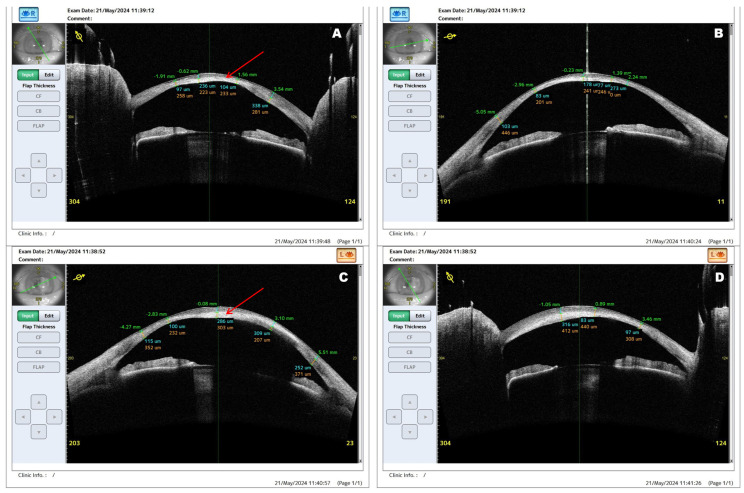
Preoperative AS-OCT imaging of bilateral lipid keratopathy. (**A**,**B**) Right eye scans obtained in different meridians show marked stromal hyperreflectivity, irregular internal stromal architecture, and increased optical density consistent with deep lipid deposition. The anterior corneal contour remains relatively preserved despite extensive stromal involvement. (**C**,**D**) Left eye scans demonstrate mid-to-deep stromal hyperreflective deposits and corneal opacification, with less pronounced structural disruption than in the right eye. Red arrows indicate hyperreflective stromal lipid deposits located within the corneal visual axis. AS-OCT helped define the depth and extent of stromal disease and supported selection of penetrating keratoplasty for full-thickness removal of visually significant opacity [1,3].

**Figure 3:**
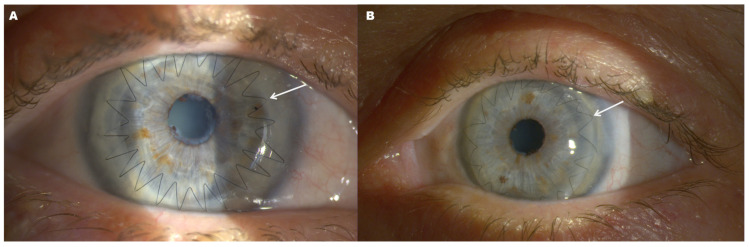
Postoperative slit-lamp photographs after staged bilateral penetrating keratoplasty. Right (**A**) and left (**B**) eyes show clear, well-centered donor grafts with visible anterior segment structures and no clinical signs of graft edema, stromal opacity, or immunological rejection. Arrows indicate the continuous 10-0 corneal suture delineating the graft–host junction and the margin of the donor corneal graft. Donor graft diameter and suture configuration were individualized according to the extent of lipid deposition and intraoperative corneal status. Residual peripheral stromal changes are minimal and spare the visual axis. Penetrating keratoplasty remains an established option in advanced lipid keratopathy with deep stromal involvement when full-thickness replacement of the affected cornea is required [1,4]. Individualized graft sizing and suture management are relevant to graft stability and postoperative corneal curvature control [3,5].

**Figure 4:**
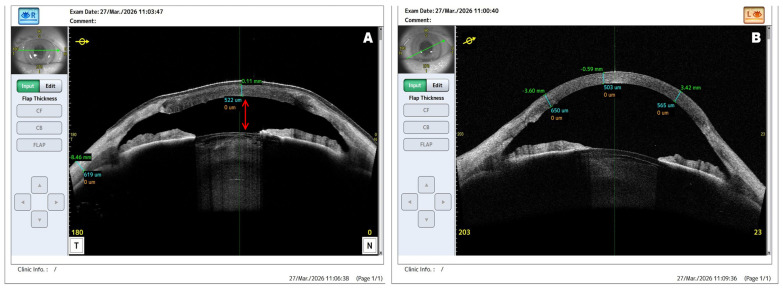
Postoperative AS-OCT imaging and detection of anterior chamber shallowing. (**A**,**B**) AS-OCT images obtained at the latest follow-up visit demonstrate well-integrated corneal grafts with preserved corneal architecture in both eyes. The graft–host interface appears regular, with no evidence of interface fluid, graft-related structural irregularity, or recurrent lipid deposition. In the right eye (**A**), the red arrow indicates anterior chamber shallowing, associated with anterior displacement of the iris–lens diaphragm. This finding was consistent with progressive lens thickening and cataract development, resulting in reduced anterior chamber depth. The patient subsequently reported a decline in BCVA in the right eye to 0.3 Snellen (0.52 logMAR), supporting the decision to proceed with planned phacoemulsification. In contrast, the left eye (**B**) demonstrates stable postoperative anterior segment anatomy with preserved anterior chamber configuration. These findings illustrate the value of AS-OCT after corneal transplantation, as it confirmed graft integrity while also identifying anterior segment changes that were not fully appreciable on slit-lamp examination and were relevant to further surgical planning [3,4]. No signs of graft rejection were observed during the 6-month follow-up, and the short-term visual prognosis is favorable given maintained graft clarity and improved BCVA. However, medium- and long-term prognosis will depend on graft stability, endothelial function, corneal response after subsequent phacoemulsification, and absence of late complications, including immunological rejection, suture-related events, endothelial decompensation, or recurrent lipid deposition.

## Data Availability

The original contributions presented in this study are included in the article. Further inquiries can be directed to the corresponding author.

## Abbreviations

The following abbreviations are used in this manuscript:

AS-OCT	Anterior Segment Optical Coherence Tomography
BCVA	Best-corrected Visual Acuity
